# Racial discrimination and anger expression: a preliminary test of a social cognitive model

**DOI:** 10.3389/fpsyg.2026.1711803

**Published:** 2026-08-12

**Authors:** Jennifer E. Graham-Engeland, Dakota D. Witzel, Emily Fair, Matthew J. Zawadzki, Melissa K. Peckins, Amish Patel, Ana Chkhaidze, Patrick Louie Robles, Michael A. Russell, Maryam Hussain, Elizabeth Brondolo

**Affiliations:** 1 Department of Biobehavioral Health, The Pennsylvania State University, University Park, PA, United States; 2 Department of Psychological Sciences, University of California, Merced, Merced, CA, United States; 3 Department of Psychology, St. John’s University, Queens, NY, United States; 4 Department of Neurology, Northwestern University, Chicago, IL, United States; 5 Jamaica Hospital Medical Center, Jamaica, NY, United States

**Keywords:** anger expression, discrimination, rumination, social and physical threat, social vigilance, negative emotions

## Abstract

**Introduction:**

Exposure to discrimination is associated with greater use of anger suppression (anger-in) and outward expression (anger-out), both of which are reflexive anger expression styles that are linked with relationship difficulties and poorer health. Social cognitive processes, such as heightened vigilance for social threats and rumination, may link discrimination to mental health, but little is known about the role of social cognition in the associations of discrimination to anger expression.

**Method:**

A racially diverse community sample (*n_analytic_* = 155, 67% women, 39% Black, 39% Latino/a, *M*_age_ = 39) self-reported racial discrimination (including two racial discrimination subtypes – stigmatization and physical threat), social vigilance, discrimination-based rumination, and discrimination-related anger expression at one time point.

**Results:**

Total (overall) discrimination was positively associated with reflexive but not reflective (anger-control) anger-expression. Tests of statistical mediation revealed significant indirect effects of rumination, but not social vigilance in the association of each type of discrimination to anger-out. Rumination also had a significant indirect effect in the relations of stigmatization to anger-in. In contrast, social vigilance demonstrated significant indirect associations between physical threat and anger-in.

**Discussion:**

Although the present work was cross-sectional and thus preliminary, findings suggest that racial discrimination is associated with anger expression styles via social cognitive factors. The pathways vary depending on the type of discrimination and the type of anger expression. Future work should include longitudinal studies to test mediation pathways. Examining social cognitive mediators of different types of discriminatory experiences to anger expression may guide the development of interventions to reduce the social costs of discrimination.

## Introduction

Discrimination is a psychosocial stressor that often evokes feelings of anger and can shape anger expression (Brondolo et al., 2009; Thayer et al., 2020). Prior work indicates that those who experience more discrimination due to race and/or ethnicity endorse higher levels of “reflexive” anger styles—including anger suppression (termed anger-in) and aggressive or a confrontational outward anger expression (i.e., anger-out; Brondolo et al., 2009; Zilioli et al., 2017). Reflexive anger expression styles are important to examine in the context of discrimination because they are linked with relationship difficulties and cardiovascular disease, both of which demonstrate stark racial disparities (Brosschot and Thayer, 1998; Lafontaine and Lussier, 2005; Williams et al., 2019).

Discriminatory experiences are common and recurrent, potentially eliciting repeated or sustained experiences of anger (Brondolo et al., 2005b; Torres and Ong, 2010). We hypothesize that anger evoked by discriminatory experiences can be prolonged by social-cognitive processes, specifically those related to social vigilance and rumination. People who experience discrimination may perceive their social environment as unsafe, leading to persistent hypervigilance around threats (Hicken et al., 2013; Mikrut et al., 2022; Sawyer et al., 2012); they may also ruminate about episodes of anger-evoking discriminatory events, prolonging the experience of anger (Borders and Wiley, 2020; Mikrut et al., 2022). Despite past work linking discrimination with social vigilance and rumination, no work to our knowledge has examined the association of these social cognitive factors with anger expression styles. Thus, the main goal of the present work was to examine how social vigilance and rumination statistically account for the link between discrimination and the use of reflexive anger styles. Because our data are cross-sectional, this investigation is preliminary. Our intention is for this work to inform the development of models that elucidate how discrimination can shape social cognitive processes that lead to anger expression, and which consequently may influence social relations.

Discrimination is a multidimensional construct, with experiences of injustice involving both social threats, including stigmatization, as well as physical threats and harassment (Abramson et al., 2015; Begay et al., 2024). Managing discriminatory experiences and their emotional sequelae can be highly challenging (Brondolo et al., 2009). For example, expressing anger (i.e., using an anger-out strategy, such as yelling or showing hostility through body language) when faced with discrimination can lead to fears of retaliation, including threats to safety or negative social outcomes (Kaiser and Miller, 2001). Expressing anger can also evoke concerns about confirming negative stereotypes about one’s group, such as stereotypes about emotionality and hostility (Dorr et al., 2007; Fitness, 2000; Park et al., 2018; Richeson and Shelton, 2007). On the other hand, anger suppression (anger-in; e.g., ignoring angry feelings or remaining silent when angry) can limit opportunities for conflict resolution and interpersonal connection in ways that undermine relationships over time. Individuals who report habitually suppressing anger tend to have poorer relationships, psychological health, and physical health (Butler et al., 2003; Chapman et al., 2013; Chervonsky and Hunt, 2017; Gross, 2002; Jorgensen and Kolodziej, 2007). Thus, effects of discrimination on anger expression may be one way in which discrimination “gets under the skin” and potentially undermines (or supports) relationships (Brondolo et al., 2012; Butler et al., 2003; Contrada et al., 2000; Renshaw et al., 2010).

Exposure to discrimination and other ethnicity-related stressors can also influence social cognition, potentially increasing vigilance and heightening attention to other social threats, including rejection or invalidation (Brondolo et al., 2005b; Broudy et al., 2007; Inzlicht et al., 2009; Kraus et al., 2011; Mikrut et al., 2022; Richeson and Shelton, 2007). Although the anticipation of threat may help individuals regulate their expectations and mitigate negative psychobiological consequences of adverse experiences (Hicken et al., 2018), remaining in a vigilant state can be costly (Hicken et al., 2018; Schmader and Johns, 2003). For example, being vigilant can reduce cognitive resources that could be utilized for other tasks or for the development of other types of coping strategies (Flanagan and Nathan-Roberts, 2019; Hicken et al., 2013; Richeson and Shelton, 2007). Maintaining vigilance also involves activation of physiological stress systems that can increase sensitivity to the hostile emotions of others (Kraus et al., 2011; Ruiz et al., 2017) and drive further reflexive anger expression. To our knowledge, however, social vigilance has not been tested as a link between discrimination and anger expression.

Experiences of discrimination have also been associated with greater proneness to rumination (Borders and Wiley, 2020). Rumination can be defined as perseverative thinking about negative, potentially distress-evoking events (Borders and Wiley, 2020; Smith and Alloy, 2009). Rumination is particularly common when the negative event is not easily understood or involves conflict or injustice that seemingly cannot be remedied (Brosschot et al., 2006; Denson, 2012). Importantly, ruminating about any stressor can prolong the duration of the experience of the stressor and intensify the overall burden of negative emotion (Gerin et al., 2012; Smyth et al., 2013). The increase in emotional burden may drive reflexive anger expression or suppression. In keeping with this possibility, rumination has been linked with more reflexive anger expression styles (e.g., Ham and You, 2018; Norma, 2017). However, the degree to which rumination can serve as a link between discrimination and anger expression also appears to be untested.

Understanding the social cognitive factors that explain relations between discrimination and anger expression may provide targets for intervention. Guided by basic research on social-cognitive factors associated with academic engagement (Oyserman et al., 2006), interventions have been successfully deployed to reduce the negative effects of discrimination on motivation and academic engagement (Birnbaum et al., 2021; Walton and Cohen, 2007). Identifying social cognitive factors that are associated with social functioning – including how they relate to anger expression evoked by race-related stress – may be valuable for the development of social cognitive interventions to support relationships (Wofford et al., 2019). This study is intended as an initial step to inform that process.

Changes to social cognition may vary depending on the context of discrimination, including the type of maltreatment. Social forms of discrimination (i.e., being treated in ways that involve exclusion or stigmatization) may activate different social cognitive processes than experiencing physical threats (Brondolo et al., 2005b) and consequently trigger different patterns of anger expression. To date, researchers have not determined if different types of discrimination are associated with anger expression via different social cognitive pathways.

Using cross-sectional data, the overarching goal of this preliminary research is to determine whether self-reported social vigilance and rumination statistically account for associations between discrimination and reflexive anger expression styles (anger-in and anger-out). We examined these effects in a diverse community sample, a majority of whom were Black or Latino/a adults. We hypothesized that (1) there would be a direct association between higher perceived discrimination overall and higher reflexive anger expression styles, and (2) social vigilance and/or rumination would account for the connection between discrimination and anger expression. The goal of these analyses was to examine unique statistical associations of both discrimination and social cognition with the specific measure of anger expression under investigation. Thus, our primary models included a measure of reflective anger expression (anger-control), as a way of controlling for the overall experience of anger and the need to engage in anger coping; further, these models accounted for the association between anger expression variables (by controlling for anger-in when examining variance in anger-out, and controlling for anger-out in analyses examining variance in anger-in). In exploratory analyses, we examined separate models using two key dimensions of discrimination – an aspect of social threat (perceived stigmatization) and physical threat (race-related threat to person or property). These analyses permitted us to determine if these varying manifestations of discrimination were differentially related to social-cognitive factors and their potential association with anger expression styles.

## Method

Participants were recruited from a university setting and a local hospital medical center in New York as part of a series of studies on the effects of racism on health (for more information on recruiment, see Keating et al., 2022; Mikrut et al., 2022). Potential participants were screened for eligibility, which involved being 18 years of age or older and having the ability to read and respond to study measures in English.

### Overall procedure

Participants in the present study (*N* = 159; *n_analytic_* = 155) provided sociodemographic information and completed well-validated self-report baseline questionnaires assessing racial discrimination, social vigilance, and discrimination-based rumination and anger expression styles. The Institutional Review Board (IRB) affiliated with the university and medical center approved the protocol, and participants were given monetary compensation.

### Measures

#### Sociodemographic variables

We assessed gender (0 = *women*, 1 = *men*), age in years, education (0 = *high school or less education*, 1 = *college or more*), household income in units of $10,000, race (dummy-coded as Black: 0 = *no*, 1 = *yes*), and ethnicity (dummy coded as Latino(a): 0 = *no*, 1 = *yes*). Individuals who identified as Latino(a) were coded as Latino(a) regardless of their racial identification.

#### Discrimination

Exposure to racial/ethnic discrimination was measured using the Brief Perceived Ethnic Discrimination Questionnaire – Community Version (PEDQ-CV; Brondolo et al., 2005a). The Brief PEDQ-CV is a 17-item scale that assesses lifetime and recent experiences of racial/ethnic discrimination in social or interpersonal contexts. Respondents indicated how often they experienced negative social experiences with items such as “Because of your ethnicity/race, have others ignored you or not paid attention to you?” Participants responded on a 5-point Likert scale ranging from 1 (*never*) to 5 (*very often*). Items were averaged together to create a “racial discrimination” score, where higher scores represent more perceived discrimination based on race/ethnicity. The Brief PEDQ-CV has been validated in a variety of communities of color, including Asian American, American Indian/Alaska Native, Latino/a, and Black samples, and demonstrated good reliability (e.g., Blair et al., 2021; Brondolo et al., 2011; Brondolo et al., 2005a). In the present sample, the reliability of this measure was excellent (*α* = 0.90).

The Brief PEDQ-CV (Brondolo et al., 2005a) yields a full-scale score and four subscale scores. Recent tests of measurement invariance across cultural/ethnic groups suggest that a four-subscale model with one larger latent variable was appropriate across groups (Arellano-Morales et al., 2015; Blair et al., 2021). Although the subscale scores assess distinct types of discrimination, they are closely associated with the total (overall) discrimination score. Given the small sample in the current study and the interest in a parsimonious model able to distinguish between social and physical race-related threat, we focused on only two dimensions: one associated with race-related social threat (the stigmatization subscale) and one associated with race-related physical threat and harassment (the threat scale). The stigmatization subscale includes items assessing the degree to which participants report being treated in a socially demeaning manner as a function of racial bias. The threat subscale includes items assessing the degree to which participants report that others have threatened or actually harmed them or their property as a function of racial bias.

#### Social vigilance

The well-validated Social Vigilance Questionnaire (SVQ; Ruiz et al., 2017) was used to assess the degree to which a person tends to engage in stress-related vigilance or monitoring of the social environment. Participants were asked, ‘In social situations…’ followed by 10 items (such as “I am cautious about what I say around others,” and “look for potential threats”) that are rated using a 5-point Likert scale ranging from 1 (*almost never*) to 5 (*almost always*). Items were averaged together such that higher scores represent more perceived social vigilance. This scale also had good reliability in the present sample (*α* = 0.90).

#### Rumination

A modified version of the rumination subscale of the Behavioral Anger Response Questionnaire (Linden et al., 2003) was used to assess ruminative tendencies related to discrimination. This assessment was adapted to include the stem information, “When you are treated badly because of your race or ethnicity…” on four items that included “I could not easily stop thinking about the event” and” I thought repeatedly about what I really would have liked to do but did not”; thus, we refer to this measure as discrimination-rumination. Participants responded using a 5-point Likert scale ranging from 1 (*not at all*) to 5 (*very much*). Items were averaged together, such that higher scores represent more discrimination-rumination. The scale had good reliability in the present sample (*α* = 0.92).

#### Anger expression

Participants completed the Spielberger Anger-Expression scales (Spielberger et al., 1995), which includes 15 questions that form 3 subscales to identify reflexive anger expression (i.e., tendencies to suppress [anger-in] or express anger with hostility [anger-out]), and reflective anger expression (i.e., anger-control). These items were adapted to include the phrase, “How do you handle the situation when you are treated badly because of your race or ethnicity?” Hence, responses reflect the tendency to use these anger expression styles in the context of perceived discrimination. Example items included “Feel angry but hold it in” (anger-in), “Show my anger in my tone of voice and my body language” (anger-out), and “Think things through before speaking” (anger-control). Participants respond using a 5-point Likert Scale ranging from 1 (*not at all*) to 5 (*very much*). Subscale items were averaged together such that higher scores for each subscale represent higher levels of anger expression. In the present sample, the subscales exhibited good reliability: anger-in (*α* = 0.79), anger-out (*α* = 0.86), anger-control (*α* = 0.80).

### Statistical analysis

#### Preliminary analyses and identification of covariates

Preliminary analyses examined whether key sociodemographic factors (age, sex, education, race, and ethnicity) were associated with any key variables in the present work (our discrimination variables, social vigilance, rumination, and anger expression styles). Although we initially considered including income as a potential covariate, almost 20% of the sample had missing data; therefore, we rely on education as our primary measure of socioeconomic status. Preliminary analyses employ ANOVA for categorical predictors and correlations for continuous variables. Demographic factors that were significantly associated with any key variables (which were age, race, and ethnicity) were then considered as potential covariates for the inferential analyses. Preliminary analyses also examined relations among the three anger-expression measures and all other key variables.

#### Inferential analyses

Given the small sample size and the need to test the hypotheses with a robust set of covariates, we used a series of PROCESS mediation analyses (SAS v2.10; Hayes, 2022) with 5,000 bootstrap samples for generating 95% percentile confidence intervals (95% bootstrap CI). As these models test one outcome (either anger-in or anger-out) at a time, we tested a series of six total models in our primary analyses. Specifically, we examined the effects of three measures of discrimination (i.e., total discrimination, stigmatization, and threat). In each analysis, we examined the indirect effects of social vigilance and rumination in the relations of discrimination to anger expression. Contrasts between the effects of the two mediators were examined for each analysis.

On an *a priori* basis, our primary analyses included the anger expression covariates. We included anger-control as a covariate in our primary models (which predicted either anger-in or anger-out), and whichever reflexive anger expression variable (anger-in or anger-out) that was not the focus of a given model (e.g., anger-out was used as a covariate in analyses of anger-in). Anger-control is the most common form of anger expression and served as a measure of the overall experience of anger and anger coping. The alternative measure of anger expression served as a means of statistically isolating the effects associated with the measure of anger expression targeted for analysis in the model.

As a sensitivity analysis, we also ran models that included no covariates, to permit examination of unadjusted associations among predictors, statistical mediators, and outcomes. In addition, we ran models that included not only the anger expression covariates, but also the sociodemographic covariates that were associated with any key study variable (age, race, and ethnicity). These were not considered our primary analyses, as the inclusion of race and ethnicity in an analysis of the effects of discrimination must be considered very cautiously, given that race and ethnicity may be confounded with the conditions that generate exposure to discrimination and shape patterns of anger-expression. Nevertheless, because the sample was socioeconomically, ethnically, and racially diverse, these additional analyses were conducted to evaluate the robustness of the observed indirect effects.

## Results

### Preliminary analyses

#### *Post-hoc* sensitivity analysis

Because of the small analytical sample size, we conducted a post-hoc sensitivity analysis to determine the effect size that would be detected with adequate power (assuming 80%; *α* = 0.05) using the multiple regression function in G*Power (Erdfelder et al., 1996). With an analytic sample size of *n* = 155 and five predictors, the study had 80% power (*α* = 0.05) to detect small effects (*f^2^* = 0.085). Notably, however, we acknowledge that this sensitivity analysis provides information on regression (e.g., direct effects) and the sample size may be relatively underpowered to detect indirect or mediational effects (Fritz and MacKinnon, 2007).

#### Sample characteristics

Of the community-dwelling participants in the present sample (*N* = 159), a final analytical sample of 155 (97.48% of full sample) had complete data on key variables and were included as the analytic sample. A total of 67.3% were women (*n* = 103, 2 missing) and a majority identified as either Black (38.71%) or Latino/a (39.35%), with a mean age of 39.26 (*SD* = 12.93; range = 18–85). About 65% of the sample had completed some college or greater education. Additional demographic details are provided in Table 1, and bivariate correlations among all key study variables are shown in Table 2.

**Table 1:** Sample demographics (*N* = 155).

Participant Characteristic	Mean (*SD*) or Percent
Race and ethnicity	
Latino/a	39.35%
Asian/Pacific Islander	4.64%
Black/African American	38.71%
White	6.45%
American Indian/Alaska Native	2.65%
Native Hawaiian	0%
Other	11.25%^a^
Age (range 18–85)	39.26 (12.93)
Women	67.32%
Income	$40,000–$50,000^b^
Education	
Grades K-8	0.66%
Grades 9–11	12.58%
High school/GED	21.85%
Some college	25.17%
Technical school	14.57%
Bachelors	15.89%
Some graduate training	1.32%
Masters or PhD	7.95%

This table provides details on sample demographic factors. ^a^The category of other was a self-disclosed racial identity and people reported the following: Caribbean/Trinidadian, East Indian, Indian, Indo-Guyanese, Mixed, Native American, and West Indian. ^b^Median income for the members of the sample who provided income data was $30,000–$40,000.

**Table 2:** Correlations among key study variables.

Variable	1.	2.	3.	4.	5.	6.	7.	8.
1. Discrimination^a^								
2. Stigma^b^	**0.82**							
3. Physical threat^b^	**0.70**	**0.41**						
4. Social vigilance	**0.31**	**0.20**	**0.35**					
5. Rumination^c^	**0.38**	**0.34**	**0.27**	**0.37**				
6. Anger-in^c^	**0.32**	**0.20**	**0.31**	**0.44**	**0.43**			
7. Anger-out^c^	**0.30**	**0.34**	0.13	**0.31**	**0.43**	**0.40**		
8. Anger-control^c^	0.12	0.07	0.07	**0.24**	**0.25**	**0.59**	**0.22**	
9. Age	**−0.20**	−0.16	−0.10	−0.14	−0.11	−0.12	−0.12	−0.09

Bolded = significant at least *p* < 0.05. *N*_analytic_ = 155, except for correlations with age (*n* = 152). ^a^Total racial discrimination, as assessed with the full racial/ethnic discrimination scale. ^b^Discrimination subscales of physical threat and stigmatization (a form of social threat). ^c^Rumination and the anger expression styles were assessed in the context of being treated badly because of race or ethnicity.

#### Sociodemographic differences in key variables

As shown in Table 2, age in years was negatively associated with total discrimination, *r*(151) = −0.20, *p* = 0.01. Age displayed negative but not significant correlations with the remaining key variables, *rs*(151) -0.09 to −0.16; *p*s range from 0.06 to 0.26. Race, ethnicity, gender, and education level differences in key variables are presented in Table 3. There were no significant gender or education level differences in scores on any key variable. Race differences were observed for anger-out, *F*(3,151) = 2.99, *p* = 0.04, anger-control, *F*(3,151) = 5.48, *p* = 0.002, and rumination, *F*(3,151) = 3.37, *p = 0.02*. Scores for Black participants were significantly higher than scores for participants identifying as “other race” for measures of anger-out and rumination. Scores of Latino(a) participants on measures of anger-out and anger-control were higher than scores of participants identifying as not Latino(a). Scores for anger-control were greater for Latino(a) than Black participants. Given that only age, race, and ethnicity had any significant associations with key study variables, we add age (in years), race (dummy coded as Black: 0 = *no*, 1 = *yes*), and ethnicity (dummy coded as Latino(a): 0 = *no*, 1 = *yes*) in order in subsequent analyses in which sociodemographic covariates were included. Gender and education were not included as covariates because they were not associated with the primary study variables.

**Table 3:** Demographic differences on key variables.

Variable	Gender		Race and ethnicity				Education	
	Male *n* = 50 Mean (*sd*)	Female *n* = 103 Mean (*sd*)	Black *n* = 60 Mean (*sd*)	Latino/a *n* = 62 Mean (*sd*)	Other Race *n* = 24 Mean (sd)	White *n* = 10 Mean (*sd*)	High School *n* = 53 Mean (*sd*)	Some College+ *n* = 97 Mean (*sd*)
Discrimination	1.66 (0.57)	1.69 (0.61)	1.76 (0.52)	1.69 (0.64)	1.45 (0.50)	1.75 (0.91)	1.69 (0.60)	1.71 (0.60)
Stigma	1.56 (0.62)	1.42 (0.56)	1.53 (0.52)	1.48 (0.62)	1.25 (0.43)	1.56 (0.88)	1.42 (0.52)	1.51 (0.62)
Threat	1.39 (0.54)	1.43 (0.70)	1.51 (0.73)	1.39 (0.54)	1.17 (0.43)	1.75 (1.00)	1.53 (0.75)	1.38 (0.60)
Anger-in	2.48 (1.00)	2.46 (1.00)	2.48 (0.97)	2.66 (0.94)^b^	2.13 (1.05)^a^	2.10 (1.16)	2.47 (1.00)	2.51 (0.99)
Anger-out	2.42 (1.04)	2.43 (1.02)	2.55 (1.01)^a^	2.55 (0.96)^a^	1.94 (1.04)^b^	2.03 (1.12)	2.38 (1.00)	2.47 (1.04)
Anger-control	2.91 (1.11)	2.95 (1.09)	2.76 (1.06)^a^	3.36 (0.93)^b^	2.60 (1.31)^a^	2.43 (1.06)^a^	2.83 (1.05)	3.05 (1.11)
Social vigilance	2.91 (1.01)	2.97 (1.00)	3.12 (1.06)	2.86 (0.96)	2.78 (0.92)	3.09 (1.02)	3.15 (1.05)	2.89 (0.98)
Rumination	2.28 (1.24)	2.59 (1.26)	2.75 (1.22)^a^	2.46 (1.23)^a^	2.26 (1.40)	1.50 (0.65)^b^	2.47 (1.25)	2.52 (1.27)

Means with different superscripts (a vs. b) are significantly different from each other, *p* < 0.05. Discrimination = Total racial discrimination; Threat and Stigma = discrimination subscales of physical threat and stigmatization (a form of social threat). Rumination and the anger expression styles were assessed in the context of being treated badly because of race or ethnicity.

#### Correlations among key variables

Unadjusted correlations between key study variables are presented in Table 2. As expected, all three anger expression variables were closely related. All types of discrimination (i.e., total discrimination, stigmatization, and threat) were significantly and positively associated with anger-in. Total discrimination and stigmatization, but not threat, were significantly positively associated with anger-out. None of the discrimination measures were associated with anger-control. Both social vigilance and rumination were significantly positively correlated with all three types of anger-expression.

### Inferential analyses

**Figure 1:**
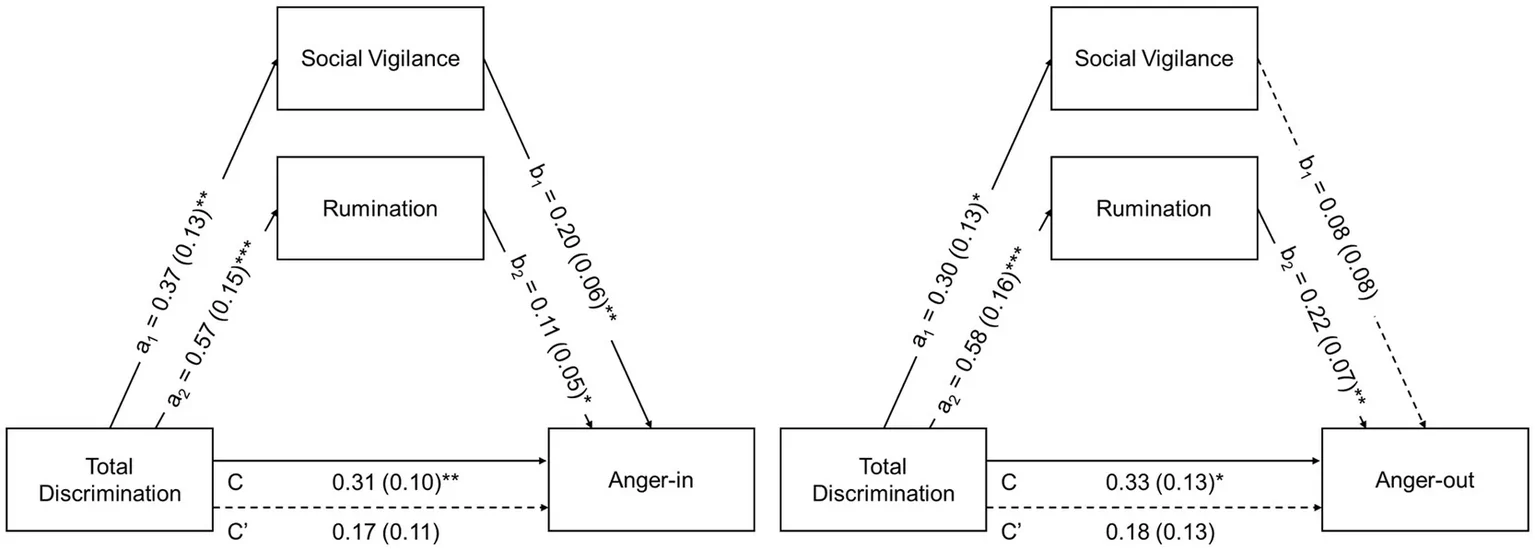
Models examining effects of total discrimination on anger expression through social vigilance and rumination. *p < 0.05, **p < 0.01, ***p < 0.001. Unstandardized estimates are presented in the figure. These models are not adjusted for covariates other than anger-control and anger-in or anger-out. Discrimination was specifically racial/ethnic discrimination, and rumination and the anger expression styles (anger-in and anger-out) were assessed in the context of being treated badly because of race or ethnicity. C represents the total effect and C′ represents the direct effect.

**Figure 2:**
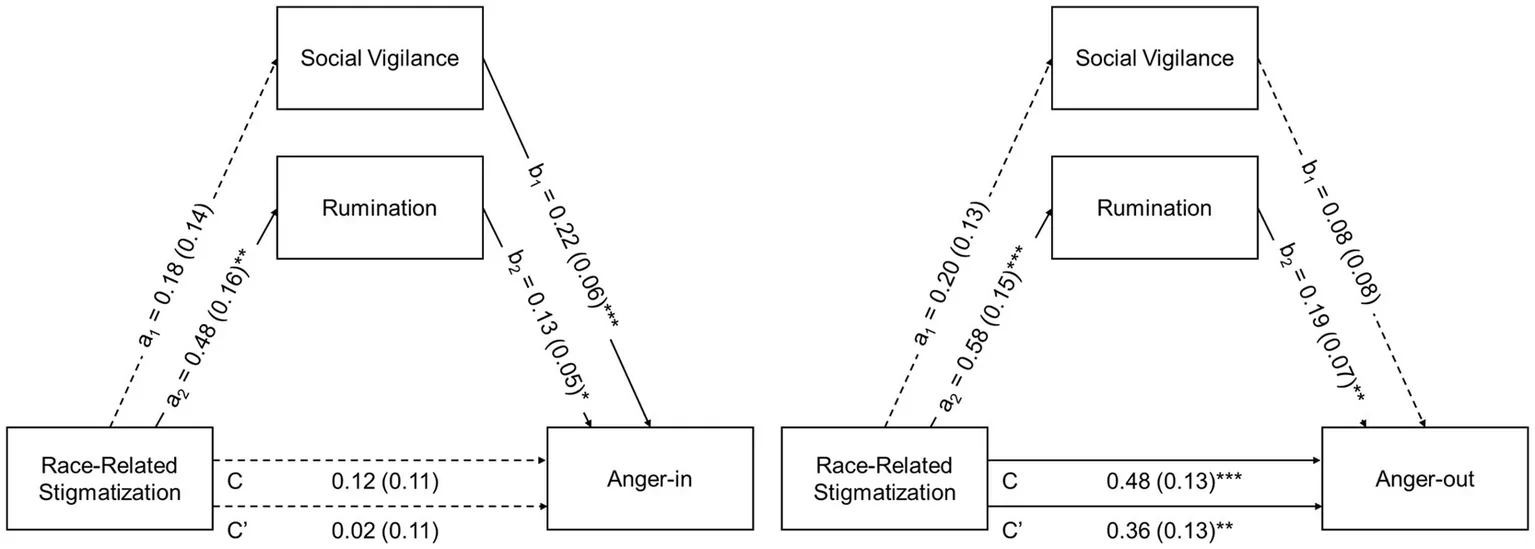
Models examining effects of the subscale of stigmatization on anger expression through social vigilance and rumination. *p < 0.05, **p < 0.01, ***p < 0.001. These models are not adjusted for covariates other than anger-control and anger-in or anger-out. Stigmatization was specifically racial/ethnic discrimination, and rumination and the anger expression styles (anger-in and anger-out) were assessed in the context of being treated badly because of race or ethnicity. C represents the total effect and C′ represents the direct effect.

**Figure 3:**
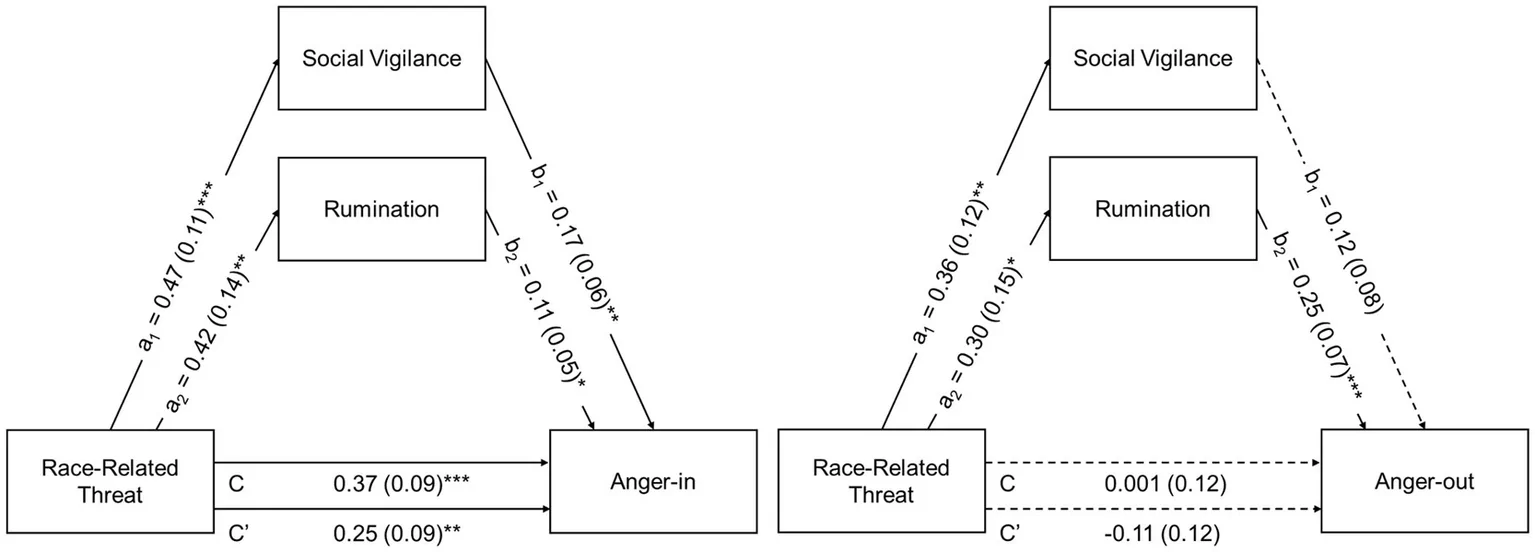
Models examining effects of the subscale of discrimination-related physical threat on anger expression through social vigilance and rumination. *p < 0.05, **p < 0.01, ***p < 0.001. These models are not adjusted for covariates other than anger-control and anger-in or anger-out. Threat was specifically racial/ethnic discrimination, and rumination and the anger expression styles (anger-in and anger-out) were assessed in the context of being treated badly because of race or ethnicity. C represents the total effect and C′ represents the direct effect.

The primary aim of the present research was to test whether social-cognitive processes, including social vigilance and discrimination-rumination, exhibit indirect effects linking perceived racial discrimination to reflexive anger expression styles (anger-in and anger-out). We report total, direct effects, and total indirect effects. However, the interpretation of the findings focused on specific indirect effects, as these represent distinct pathways in multiple mediator models. Tables 4–6 provide the indirect effects of social vigilance and rumination on the association between total discrimination (Table 4), discrimination-related stigma (Table 5), and discrimination-related physical threat (Table 6) and the anger expression outcomes. Coefficients for relations among variables in our primary analyses are displayed in Figures 1–3. Supplementary Figures 1–6 present findings from the sensitivity analyses (unadjusted and full covariate adjusted models).

**Table 4:** Indirect effects of social vigilance and rumination in the relationship of total discrimination to anger-in and anger-out.

Variable	Anger-in		Anger-out	
	Indirect Effect (ab)	Bootstrapped 95% CI	Indirect Effect (ab)	Bootstrapped 95% CI
Models with anger expression covariates				
Total	0.14	[0.04, 0.23]	0.15	[0.04, 0.33]
Social vigilance	0.08	[0.01, 0.15]	0.03	[−0.02, 0.11]
Rumination	0.06	[−0.002, 0.13]	0.12	[0.03, 0.28]
Contrast	0.01	[−0.07, 0.11]	−0.10	[−0.25, 0.02]
Sensitivity models with no covariates				
Total	0.33	[0.18, 0.50]	0.29	[0.13, 0.54]
Social Vigilance	0.15	[0.05, 0.28]	0.08	[−0.01, 0.23]
Rumination	0.17	[0.06, 0.31]	0.21	[0.08, 0.40]
Contrast	−0.02	[−0.20, 0.17]	−0.14	[−0.34, 0.05]
Sensitivity models with anger expression and demographic covariates				
Total	0.12	[0.03, 0.21]	0.13	[0.03, 0.30]
Social Vigilance	0.06	[−0.003, 0.13]	0.02	[−0.02, 0.10]
Rumination	0.06	[−0.00, 0.13]	0.11	[0.02, 0.26]
Contrast	0.002	[−0.09, 0.09]	−0.09	[−0.24, 0.02]

For models with anger-in as the outcome, anger expression covariates include anger-out and anger-control. For models with anger-out as the outcome, anger expression covariates include anger-in and anger-control. Demographic covariates were age, race, and ethnicity.

**Table 5:** Indirect effects of social vigilance and rumination in the relationship of race-related stigmatization to anger-in and anger-out.

Variable	Anger-in		Anger-out	
	Indirect effect (ab)	Bootstrapped 95% CI	Indirect effect (ab)	Bootstrapped 95% CI
Models with anger expression covariates				
Total	0.10	[−0.01, 0.21]	0.13	[0.03, 0.28]
Social vigilance	0.04	[−0.04, 0.11]	0.02	[−0.01, 0.08]
Rumination	0.06	[0.01, 0.13]	0.11	[0.02, 0.25]
Contrast	−0.02	[−0.10, 0.07]	−0.09	[−0.23, 0.01]
Sensitivity models with no covariates				
Total	0.29	[0.12, 0.48]	0.23	[0.09, 0.46]
Social Vigilance	0.11	[0.01, 0.24]	0.05	[−0.003, 0.17]
Rumination	0.18	[0.07, 0.33]	0.18	[0.06, 0.36]
Contrast	−0.07	[−0.23, 0.10]	−0.13	[−0.30, 0.02]
Sensitivity models with anger expression and demographic covariates				
Total	0.09	[−0.02, 0.19]	0.11	[0.02, 0.26]
Social vigilance	0.03	[−0.05, 0.10]	0.01	[−0.01, 0.07]
Rumination	0.06	[0.005, 0.13]	0.10	[0.01, 0.24]
Contrast	−0.03	[−0.12, 0.05]	−0.09	[−0.22, 0.01]

For models with anger-in as the outcome, anger expression covariates include anger-out and anger-control. For models with anger-out as the outcome, anger expression covariates include anger-in and anger-control. Demographic covariates were age, race, and ethnicity.

**Table 6:** Indirect effects of social vigilance and rumination in the relationship of race-related threat to anger-in and anger-out.

Variable	Anger-in		Anger-out	
	Indirect effect (ab)	Bootstrapped 95% CI	Indirect effect (ab)	Bootstrapped 95% CI
Models with anger expression covariates				
Total	0.13	[0.05, 0.21]	0.12	[0.03, 0.24]
Social Vigilance	0.08	[0.02, 0.14]	0.04	[−0.01, 0.11]
Rumination	0.04	[0.003, 0.09]	0.07	[0.01, 0.19]
Contrast	0.04	[−0.04, 0.11]	−0.03	[−0.16, 0.07]
Sensitivity models with no covariates				
Total	0.27	[0.16, 0.40]	0.26	[0.13, 0.42]
Social Vigilance	0.15	[0.06, 0.25]	0.10	[0.01, 0.22]
Rumination	0.12	[0.04, 0.22]	0.16	[0.07, 0.30]
Contrast	0.03	[−0.11, 0.17]	−0.06	[−0.24, 0.11]
Sensitivity models with anger expression and demographic covariates				
Total	0.11	[0.03, 0.19]	0.10	[0.02, 0.21]
Social vigilance	0.07	[0.01, 0.13]	0.04	[−0.02, 0.10]
Rumination	0.04	[0.002, 0.09]	0.06	[0.001, 0.16]
Contrast	0.03	[−0.05, 0.10]	−0.03	[−0.14, 0.07]

For models with anger-in as the outcome, anger expression covariates include anger-out and anger-control. For models with anger-out as the outcome, anger expression covariates include anger-in and anger-control. Demographic covariates were age, race, and ethnicity.

#### Total discrimination to anger-in

In our primary analyses, shown in Figure 1 and Table 4, the ‘*a’* paths from total discrimination to both social vigilance and rumination were significant, as were the ‘*b’* paths from social vigilance and rumination to anger-in. In our primary analyses (with anger expression covariates included), the total effects were significant, but the direct effects were not, and the total indirect effects were significant. Only the indirect effects of social vigilance, but not rumination were significant.

In the sensitivity analyses, when no covariates were included in the model, the indirect effects of both social vigilance and rumination in the relations of total discrimination to anger-in were significant (see Table 4 and Supplementary Figure 1). In the supplementary sensitivity analyses including all covariates (i.e., anger expression, age, race, and ethnicity), the indirect effects of social vigilance and rumination were no longer significant (see Table 4 and Supplementary Figure 2). In both the sensitivity and covariate adjusted models, the total effects were significant, the direct effects were not, and the total indirect effects were significant.

#### Total discrimination to anger-out

In the primary analyses, shown in Figure 1 and Table 4, the *‘a’* paths from total discrimination to both social vigilance and rumination were significant. The ‘*b’* paths from rumination to anger-out were significant, but the ‘*b’* paths from social vigilance to anger-out were not. The total effects but not the direct effects were significant. The total indirect effects were significant, as were the indirect effect of rumination but not the indirect effect of social vigilance. The contrast between the two mediators was not significant.

These patterns were unchanged in sensitivity analyses with no covariates (see Table 4 and Supplementary Figure 1), and in the fully-adjusted analyses including all covariates (see Table 4 and Supplementary Figure 2).

#### Stigmatization to anger-in

In the primary analyses, shown in Figure 2 and Table 5, the ‘*a’* path from stigmatization to rumination was significant, but the ‘*a’* path from stigmatization to social vigilance was not. The ‘*b’* paths from both social vigilance and rumination to anger-in were significant. Neither the total nor the direct effects were significant. The total indirect effects also were not significant, but the indirect effect through rumination in the association of stigmatization to anger-in was significant.

In the sensitivity analyses (with no covariates included), the total effects were significant, but the direct effects were not. The total indirect effects were significant, as were both the indirect effects through rumination and social vigilance (See Table 5 and Supplementary Figure 3). In the fully adjusted analyses, as was the case for the primary analyses, neither the total nor direct effects were significant. The total indirect effect was also not significant, but the indirect effect through rumination remained significant (see Table 5 and Supplementary Figure 4).

#### Stigmatization to anger-out

In the primary analyses shown in Table 5 and Figure 2, the ‘*a’* path from stigmatization to rumination was significant, but the ‘*a’* path from stigmatization to social vigilance was not. The ‘*b’* path from rumination to anger-out was significant, but the ‘*b’* pathway from social vigilance to anger-out was not. The total effect and the direct effects were significant. The total indirect effect was significant, as was the indirect effect through rumination, but the indirect effect through social vigilance was not.

In the sensitivity analyses with no covariates, as shown in Table 5 and Supplementary Figure 3, the total and direct effects were significant. The total indirect effect was also significant. The indirect effect through rumination, but not social vigilance was significant. In the fully adjusted analyses (shown in Table 5 and Supplementary Figure 4), as was the case for the primary analyses, both the total and direct effects were significant. The total indirect effect was significant, as was the indirect effect of rumination but not the indirect effect of social vigilance.

#### Physical threat to anger-in

In the primary analyses, as shown in Table 6 and Figure 3, the ‘*a’* paths from physical threat to rumination and to social vigilance were significant. The ‘*b’* paths from both social vigilance and rumination to anger-in were significant. The total effects and direct effects of physical threat on anger-in were significant. The total indirect effects and the indirect effects of both social vigilance and rumination were significant.

In the sensitivity analyses with no covariates, as shown in Table 6 and Supplementary Figure 5, the total effect was significant, but the direct effect was not. The total indirect effect and the indirect effects through both rumination and social vigilance were significant. In the fully adjusted analyses (shown in Table 6 and Supplementary Figure 6), the pattern of results remained the same as the primary analyses, with significant indirect effects through both rumination and social vigilance.

#### Physical threat to anger-out

In the primary analyses, as shown in Table 6 and Figure 3, the ‘*a’* paths from physical threat to rumination and social vigilance were significant. The ‘*b’* pathway from rumination to anger-out was significant, but the ‘*b’* pathway from social vigilance to anger-out was not. Neither the total effects nor direct effects of threat on anger-out were significant, but the total indirect effects were significant. The indirect effects of rumination, but not social vigilance were significant.

In the sensitivity analysis with no covariates, shown in Table 6 and Supplementary Figure 5, neither the total nor direct effects were significant. The total indirect effects were significant, as were the indirect effects through *both* rumination and social vigilance. The results for the fully adjusted analyses (see Table 6 and Supplementary Figure 6), were not different from the results of the primary analyses. The total indirect effects were significant, as were the indirect effects of rumination, but not social vigilance.

## Discussion

We aimed to investigate associations between discrimination and reflexive anger expression styles (anger-in and anger-out) and to examine whether these associations were linked to social cognitive factors, specifically social vigilance and discrimination-related rumination. We conceptualized social vigilance and rumination as processes that may be associated with responses to discrimination-related stressors and, in turn, with patterns of anger expression. The goal of these preliminary analyses was to further develop social cognitive models of the associations between discrimination and emotional responses (Brondolo et al., 2018; Mikrut et al., 2022). Our intention is for these models to serve as proof-of-concept and to inform future investigations of the role of discrimination in social and health disparities.

In line with prior work reporting that discrimination was associated with both reflexive anger expression styles (Brondolo et al., 2005b), all three measures of discrimination examined in the present work (total discrimination and subscales of stigmatization and physical threat) were positively correlated with anger-in, and total discrimination and stigmatization were associated with anger-out. Primary analyses in the present work controlled for reflective anger expression (i.e., anger control) – which was itself not associated with our discrimination measures but which was associated with anger-in and anger-out as well as social vigilance and rumination – and focused on whether there were indirect effects on both anger-in and anger-out via social vigilance and rumination. In aggregate, findings supported the overall hypothesis that there are indirect effects involving social cognitive processes (i.e., social vigilance and rumination) in the association between discrimination and reflexive anger expression.

### Social cognitive pathways

Significant indirect effects of rumination were observed in analyses of discrimination predicting anger-out. The indirect effect of rumination on anger-out was reduced to non-significance in the total discrimination model after inclusion of demographic covariates (age, race, and ethnicity) but was robust to demographic covariates in models using stigmatization or threat as predictors. There were also significant indirect effects of rumination in the association between discriminatory stigmatization and threat (but not total discrimination) on anger-in.

The evidence for indirect effects through social vigilance was more variable and dependent on model specification than the indirect effect through rumination. Social vigilance did not show significant indirect effects in analyses of total discrimination, stigmatization, or threat predicting anger-out. However, social vigilance was associated with a significant indirect effect in analyses of both total discrimination and threat predicting anger-in. The indirect effects of social vigilance in the association between threat and anger-in remained significant after inclusion of demographic covariates. In contrast, the indirect effect of social vigilance in the association between total discrimination and anger-in was not significant when demographic variables were included.

Rumination, or perseverative cognition, may serve to prolong and intensify anger (Gerin et al., 2012), consequently potentiating reflexive anger expression. The pattern of findings on the indirect effects of rumination is consistent with strong evidence that those who report more experiences of stigmatization are likely to ruminate about these experiences (Hatzenbuehler et al., 2009), and this rumination may exacerbate emotional distress (Gerin et al., 2006) and drive reflexive anger coping. In contrast, social vigilance may increase the individual’s awareness of potential threats and thereby increase the likelihood that the individual will reflexively suppress anger to avoid potential retaliation. Notably, the vigilance-avoidance theory suggests that higher levels of vigilance are associated with an initial increase in attention to threat, followed by attentional and emotional avoidance (Derakshan et al., 2007) – avoidance which may be accompanied by suppression of feelings, including anger.

Discrimination-related physical threats specifically were linked with anger-in via both discrimination-rumination and social vigilance. In the context of race-related physical threat, it is possible that vigilance may potentiate greater awareness of the threatening nature of the situation and motivate suppression rather than active confrontation to reduce retaliation (Derakshan et al., 2007; Rostosky et al., 2022). Although confrontation coping may have benefits (e.g., Czopp et al., 2006; Wood et al., 2025), targeted individuals may not perceive these benefits as outweighing the costs in a physically threatening situation and may rely on vigilance to keep them safe in a complex and dangerous situation.

It is important to note that the pattern of indirect effects observed in the total discrimination model to anger-in analysis was attenuated when race and ethnicity were included as covariates. This pattern highlights the complex relationships among race, discrimination exposure, and anger expression. Race and ethnicity may function as antecedents that shape the likelihood and nature of exposure to discrimination. In this sense, statistically controlling for race may partially adjust for structural and contextual processes that give rise to discrimination experiences themselves. In addition, race may exert effects on anger-in through mechanisms that extend beyond directly perceived interpersonal discrimination, including broader social or contextual factors associated with racialized experiences.

### Broader perspectives and future directions

These findings represent cross-sectional associations and will require replication with different methods (e.g., longitudinal and/or ecological momentary assessment designs). However, results provide an initial understanding of how discrimination may ultimately undermine relational well-being and highlight the value of further studies of the ways in which the association of discrimination to social cognition and anger expression may undermine social relations.

Prior research has documented the social costs of confrontation or anger-out (e.g., Monteith et al., 2025). However, it is important to note that not all consequences of anger expression (even reactive and/or aggressive forms) are negative (Averill, 1983; Kassinove and Sukhodolsky, 1995). For example, anger expression can be useful in establishing or reinforcing boundaries in social relationships (Karppinen et al., 2023). In some contexts, outward anger expression (even when expressed in hostile or aggressive ways) may serve as a form of advocacy (Czopp et al., 2006; Monteith et al., 2025). In the context of discrimination, the expression of anger may serve as an important signal to others that the targeted individual is aware they are being treated unfairly, and this awareness might trigger positive change in both parties that reduces exposure over time (Czopp et al., 2006). Confronting racial discrimination can lead to positive consequences, including increased self-esteem, mood, and autonomy for the targeted individual (Wood et al., 2025), changes in others’ behavior (Monteith et al., 2022), or longer-term social change (Czopp et al., 2006; van Zomeren et al., 2008). However, the effects of anger expression on social relations are complex, and the outcomes may vary depending on the targeted individual’s prior experiences with discrimination or other contextual factors (Wofford et al., 2019). For example, a recent daily diary study found that although the use of anger-out was associated with subsequent social interactions that were more negative, the consequences of outward anger expression were less negative for those who reported high (versus low) levels of exposure to discrimination (Dial et al., 2025).

Frequent use of either anger suppression or aggressive, outward anger expression may undermine social capital by straining bonds of connection (Brondolo et al., 2012; Butler et al., 2003; Renshaw et al., 2010). Future work following individuals over time to capture momentary experiences of different types of emotional expression will be valuable to determine how such patterns play out in daily life. Research is also needed to understand the interpersonal, sociocultural, and environmental boundaries around which anger expression and suppression are adaptive and valuable versus problematic (Dial et al., 2025).

Anger expression patterns driven by social vigilance and rumination may serve to maintain the stress associated with discrimination. Understanding the ways in which different types of unfair treatment influence vigilance and rumination may help clarify the ways in which discrimination affects social relations. Interventions focused on addressing these social-cognitive sequelae of discrimination could help targeted individuals develop greater flexibility in use of coping strategies or self-protective behaviors (Brown et al., 2011).

Greater awareness of the relationship of discrimination to both social-cognitive processes and anger expression may help to change the dialogue about race, discrimination, and anger. A broader understanding of how discrimination is experienced can be empowering (Begay et al., 2024). Specifically, presenting detailed information about subtypes of discriminatory experiences may facilitate faster recognition of actions that may be motivated by bias. This awareness can reduce the cognitive burden on targeted individuals (Richeson and Shelton, 2007). Further, if it can be established that rumination and social vigilance are downstream effects of different types of discrimination, this information might prompt allies to better recognize the burden on targeted individuals. Allies can join in discussing collaborative actions that can reduce the need for such vigilance and interrupt deleterious rumination (e.g., by offering targeted and observable support).

Similarly, if future longitudinal or experimental research supports the role of discrimination in shaping social cognition and anger expression, such findings could have implications for public health interventions focused on transforming social norms, an approach with documented success in many contexts (see Cislaghi and Berkowitz, 2021; Hildebrand et al., 2024). These and other public health education approaches can foster broader discussion about the best ways to directly address and potentially modify both the targets’ and allies’ views of the value of expressing anger and confronting others when discriminatory behavior occurs.

### Limitations

The results of the present research should be considered preliminary. First, analyses were based on self-report measures, including measures of anger expression styles, which can reflect global perceptions of the self that may or may not reflect behavior in daily life. As noted, future studies with behavioral or momentary measures are needed to clarify whether individuals who experience discrimination tend to suppress anger and/or express anger in confrontational ways in daily life, and to determine if these anger expression patterns follow acute or prolonged episodes of vigilance and/or rumination.

Further, analyses were cross-sectional, making it impossible to determine causal directionality from the present findings. Although our models were driven by extant literature and theory, there are likely bi-directional relationships between the key constructs studied here. For example, individuals who express anger reflexively may be subjected more frequently to unfair treatment (Park et al., 2018), which in turn may prompt more suppression. It is also possible that there are constructs unmeasured in the current research (e.g., depression or emotion regulation more broadly) that may help explain the observed key relationships among discrimination, rumination and social vigilance, and anger expression styles. It would be valuable for future studies to examine the relations among these constructs using a longitudinal approach, and ideally via a design that allows for assessment of the constructs in an ecologically valid manner, enabling participants to rely less on their recall of past events and helping to eliminate recall bias. Finally, our focus was on examining responses to discrimination, and thus we did not measure the degree to which discrimination was associated with social cognition and anger expression evoked in other contexts.

## Conclusion

The present research found significant associations between perceived discrimination and the reflexive anger expression styles of anger-in and anger-out and identified social cognitive factors – social vigilance and discrimination-rumination – that displayed indirect effects in these relations. Rumination displayed a significant indirect effect in the relations of stigmatization and threat to anger-out; in contrast, social vigilance demonstrated significant indirect effects in the association of threat to anger-in. To the extent that such patterns observed in the present research are ongoing and chronic, they may help explain how discrimination can perpetuate a vicious cycle that may contribute to health disparities. Those who experience more discrimination may be at risk for disruptions to social relationships, adding to the burden of psychosocial stress and undermining support (Wofford et al., 2019). Continuing to evaluate the effects of different types of discriminatory exposure will be valuable, because mechanisms linking discrimination to outcomes appear to vary by types of exposure. Future research will be crucial before conclusions about causality can be drawn, but present findings are in line with the possibility that social cognitive processes may play a role (Brondolo et al., 2018; Mikrut et al., 2022). The present preliminary findings can be viewed as a framework to guide researchers as they develop and test interventions aimed at mitigating the interpersonal and emotional consequences of exposure to discrimination.

## Data Availability

The data used in this study were part of a larger project conducted under the Collaborative Health Integration Research Program (CHIRP). These data are not publicly available due to privacy or ethical restrictions, but may be made available to qualified researchers upon reasonable request. Interested individuals should contact the Principal Investigator of CHIRP, Elizabeth Brondolo, at brondole@stjohns.edu.
