# Supplementary material for: Racial discrimination and anger expression: a preliminary test of a social cognitive model
Source: Front Psychol. 2026 Aug 12;17:1711803. doi: 10.3389/fpsyg.2026.1711803 (PMC13506903; doi:10.3389/fpsyg.2026.1711803)
Supplement: Supplementary file 1 [file Data_Sheet_1.pdf]

Supplemental Figure 1

Unadjusted Models Examining Effects of Total Discrimination on Anger Expression Through Social Vigilance and Rumination

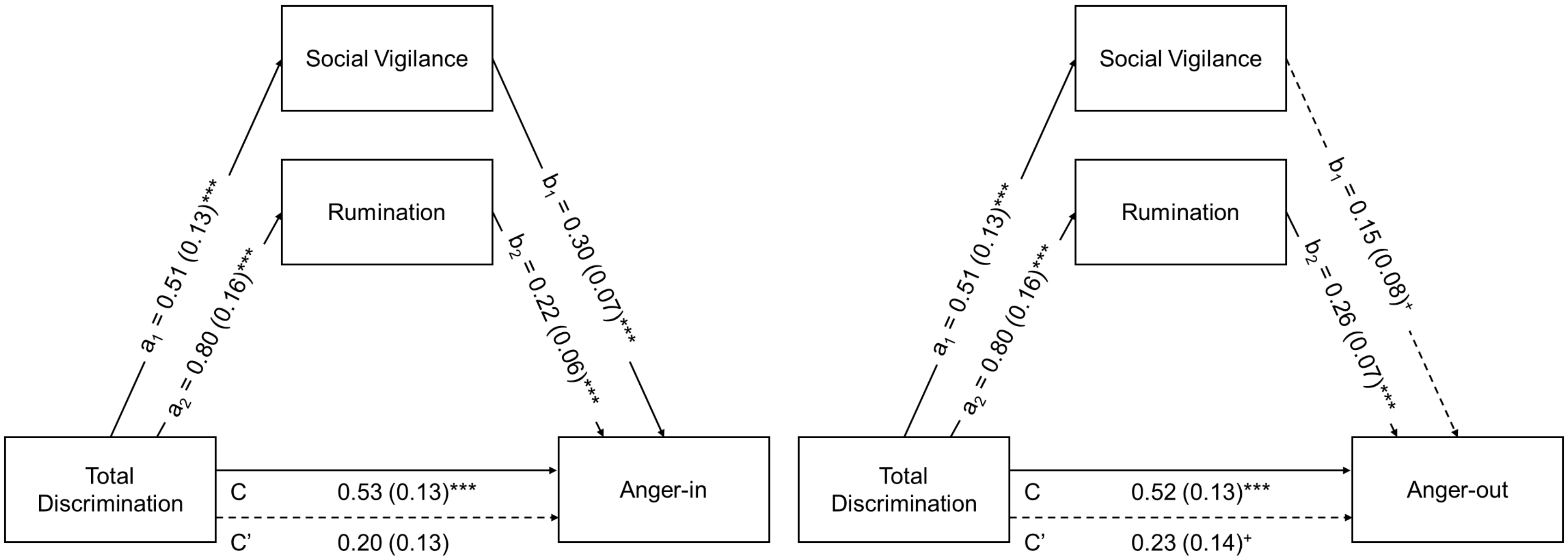

Note. $^+$ *p* < .10, * *p* < .05, ** *p* < .01, *** *p* < .001.

These models are not adjusted for covariates. Discrimination was specifically racial/ethnic discrimination, and rumination and the anger expression styles (anger-in and anger-out) were assessed in the context of being treated badly because of race or ethnicity. C represents the total effect and C’ represents the direct effect.

Supplemental Figure 2

Covariate Adjusted Models Examining Effects of Total Discrimination on Anger Expression Through Social Vigilance and Rumination

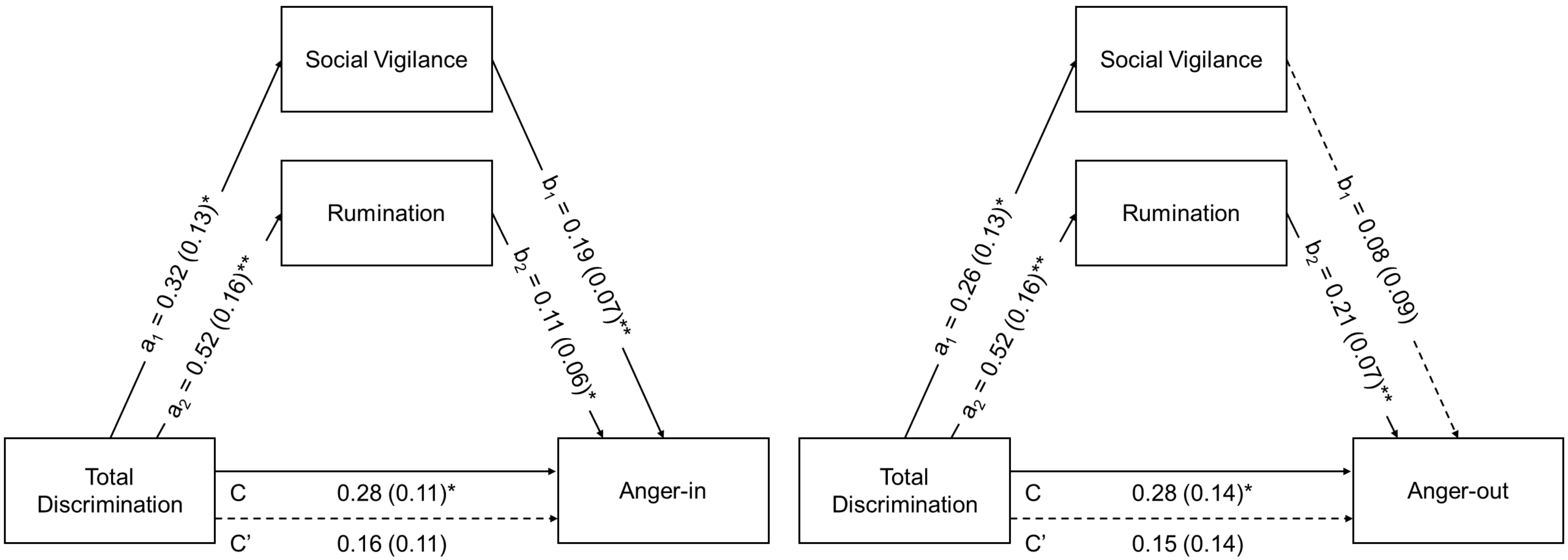

Note. * $p < .05$, ** $p < .01$, *** $p < .001$.

These models are adjusted for anger-control, anger-in or anger-out, age, race, and ethnicity. Discrimination was specifically racial/ethnic discrimination, and rumination and the anger expression styles (anger-in and anger-out) were assessed in the context of being treated badly because of race or ethnicity. C represents the total effect and C’ represents the direct effect.

Supplemental Figure 3

Unadjusted Models Examining Effects of Race-related Stigmatization on Anger Expression Through Social Vigilance and Rumination

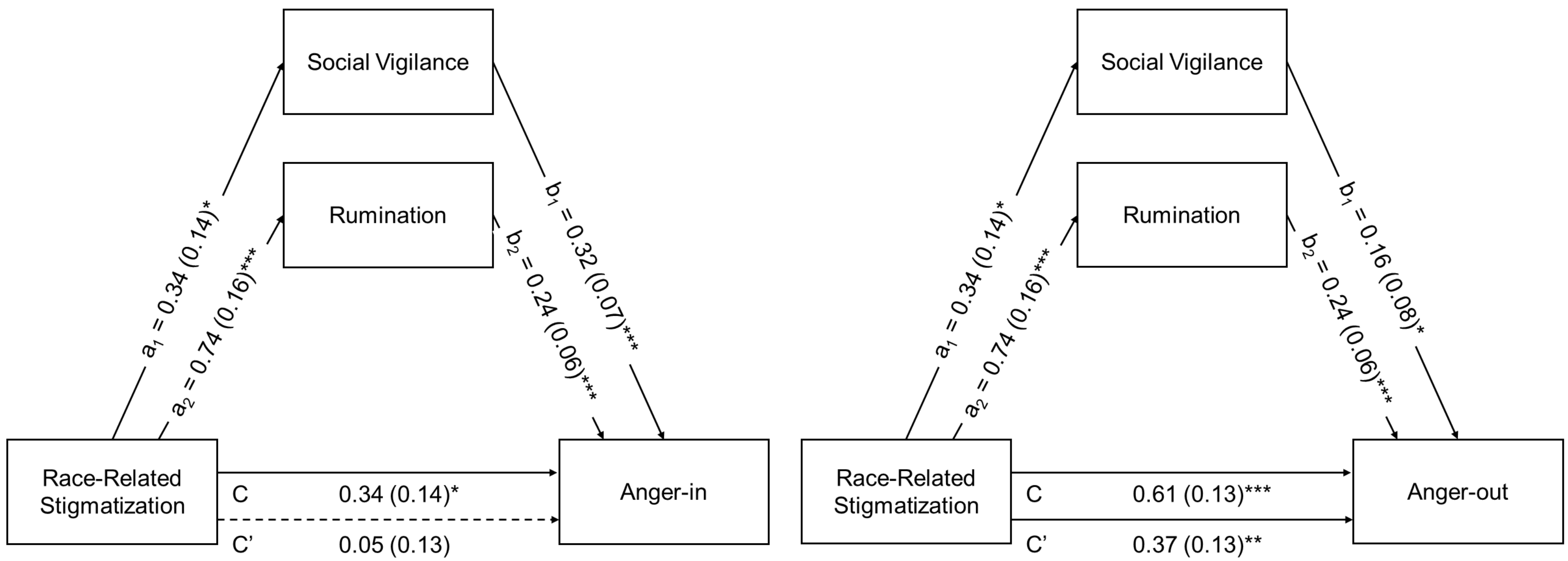

Note. * $p < .05$, ** $p < .01$, *** $p < .001$.

These models are not adjusted for covariates. Stigmatization was specifically racial/ethnic discrimination, and rumination and the anger expression styles (anger-in and anger-out) were assessed in the context of being treated badly because of race or ethnicity. C represents the total effect and C' represents the direct effect.

Supplemental Figure 4

Covariate Adjusted Models Examining Effects of Race-related Stigmatization on Anger Expression Through Social Vigilance and Rumination

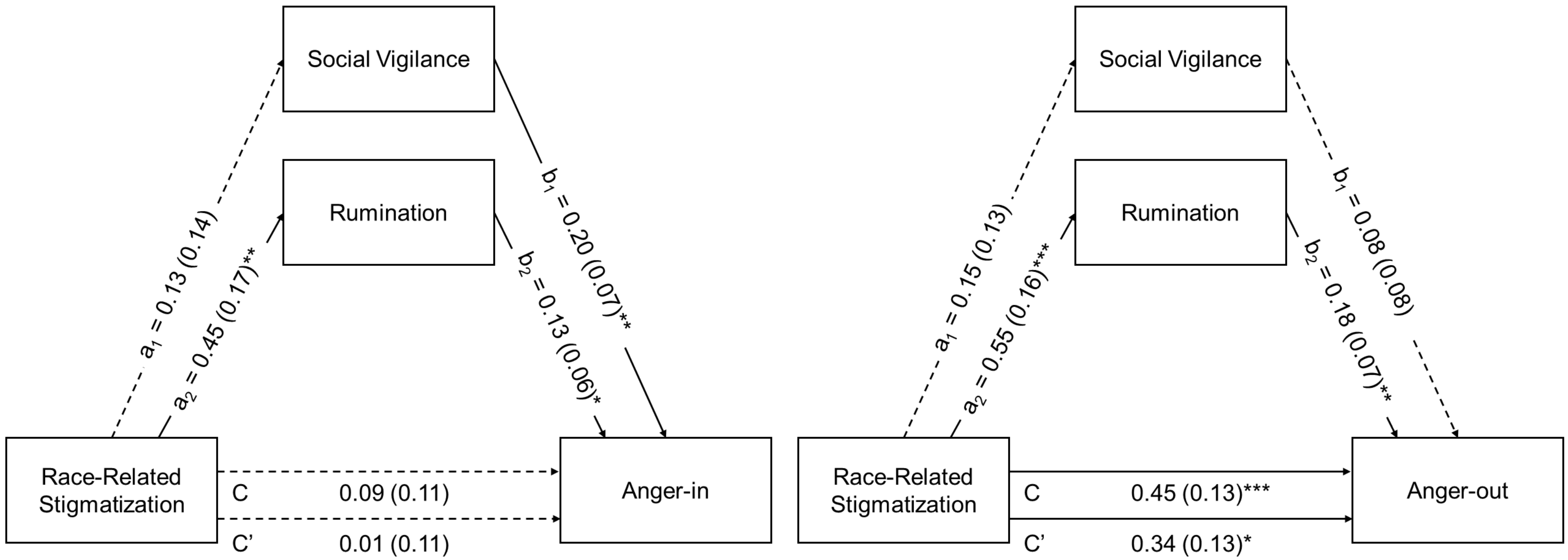

Note. * $p < .05$, ** $p < .01$, *** $p < .001$.

These models are adjusted for anger-control, anger-in or anger-out, age, race, and ethnicity. Stigmatization was specifically racial/ethnic discrimination, and rumination and the anger expression styles (anger-in and anger-out) were assessed in the context of being treated badly because of race or ethnicity. C represents the total effect and C' represents the direct effect.

Supplemental Figure 5

Unadjusted Models Examining Effects of Race-related Threat on Anger Expression Through Social Vigilance and Rumination

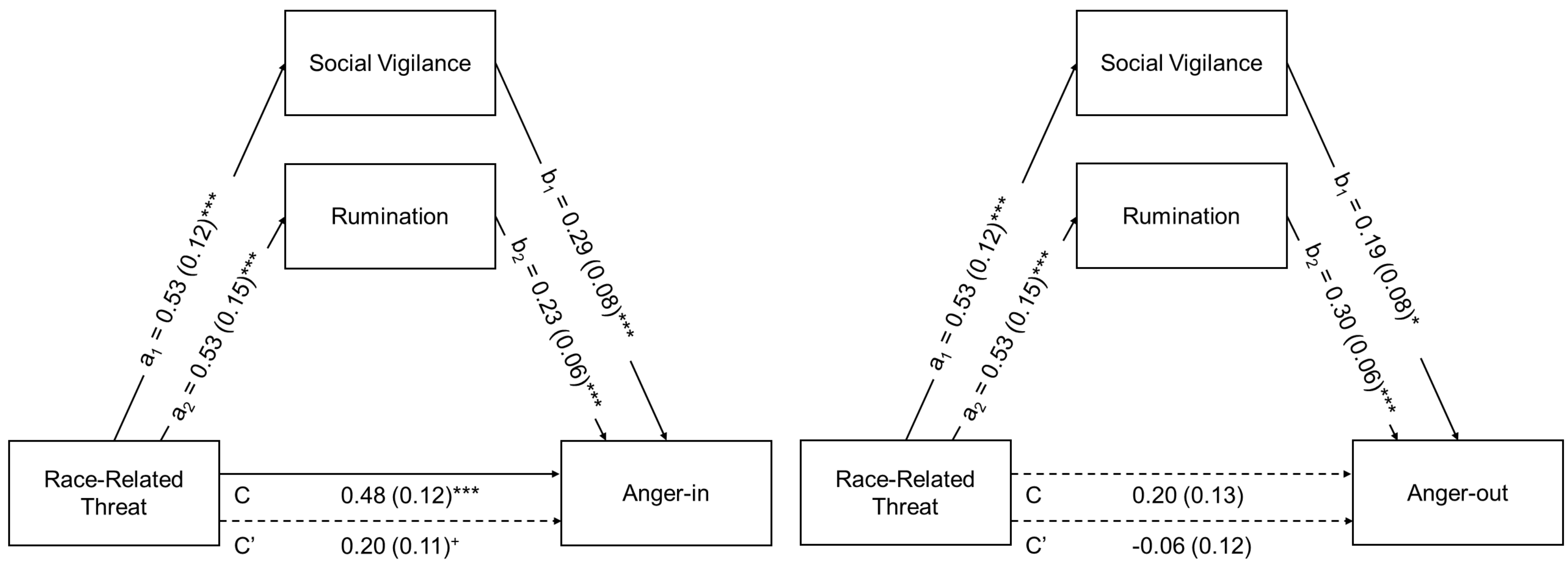

Note. $^{+}$ *p* < .10, * *p* < .05, ** *p* < .01, *** *p* < .001.

These models are not adjusted for covariates. Threat was specifically racial/ethnic discrimination, and rumination and the anger expression styles (anger-in and anger-out) were assessed in the context of being treated badly because of race or ethnicity. C represents the total effect and C' represents the direct effect.

Supplemental Figure 6

Covariate Adjusted Models Examining Effects of Race-related Threat on Anger Expression Through Social Vigilance and Rumination

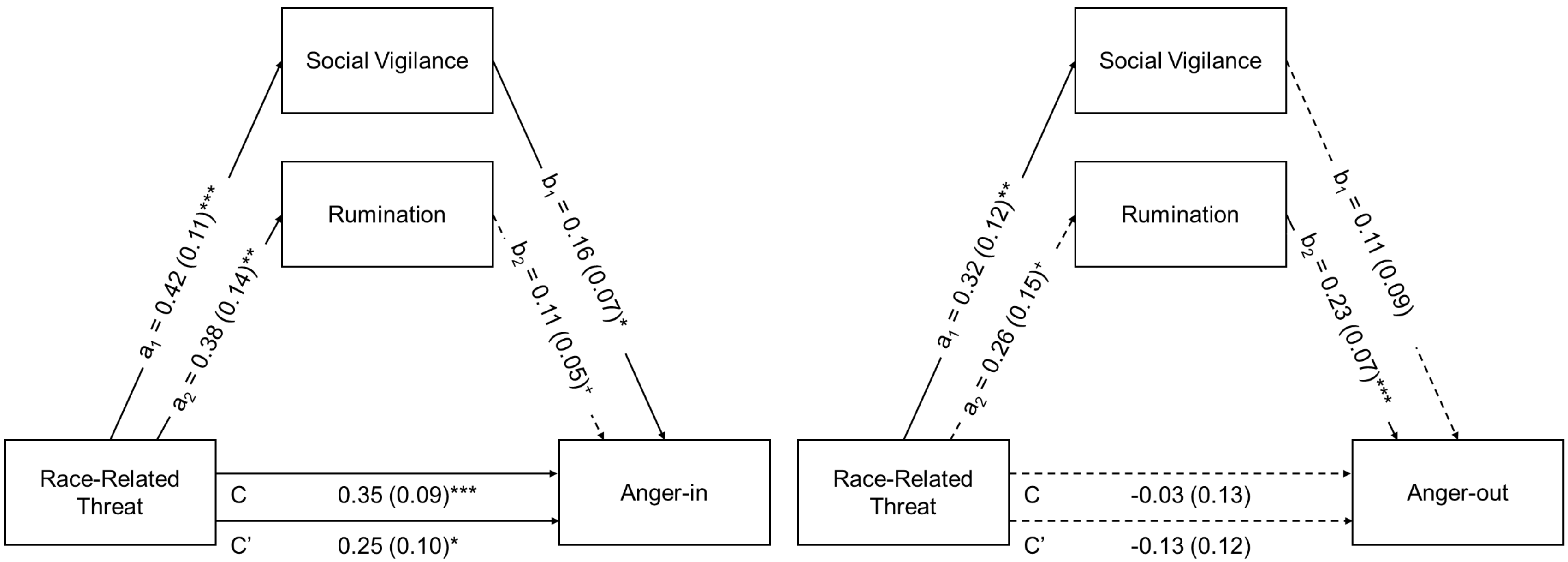

Note. $^{+}$ *p* < .10, * *p* < .05, ** *p* < .01, *** *p* < .001.

These models are adjusted for anger-control, anger-in or anger-out, age, race, and ethnicity. Threat was specifically racial/ethnic discrimination, and rumination and the anger expression styles (anger-in and anger-out) were assessed in the context of being treated badly because of race or ethnicity. C represents the total effect and C’ represents the direct effect.
